# Supplementary material for: Gene regulatory networks controlling differentiation, survival, and diversification of hypothalamic Lhx6-expressing GABAergic neurons
Source: Commun Biol. 2021 Jan 21;4:95. doi: 10.1038/s42003-020-01616-7 (PMC7820013; doi:10.1038/s42003-020-01616-7)
Supplement: Supplementary file 1 — Supplementary Information [file 42003_2020_1616_MOESM1_ESM.pdf]

Supplementary Information

Gene regulatory networks controlling differentiation, survival, and diversification of hypothalamic Lhx6-expressing GABAergic neurons.

Dong Won Kim, Kai Liu, Zoe Qianyi Wang, Yi Stephanie Zhang, Abhijith Bathini, Matthew P Brown, Sonia Hao Lin, Parris Whitney Washington, Changyu Sun, Susan Lindtner, Bora Lee, Hong Wang, Tomomi Shimogori, John L.R. Rubenstein, Seth Blackshaw

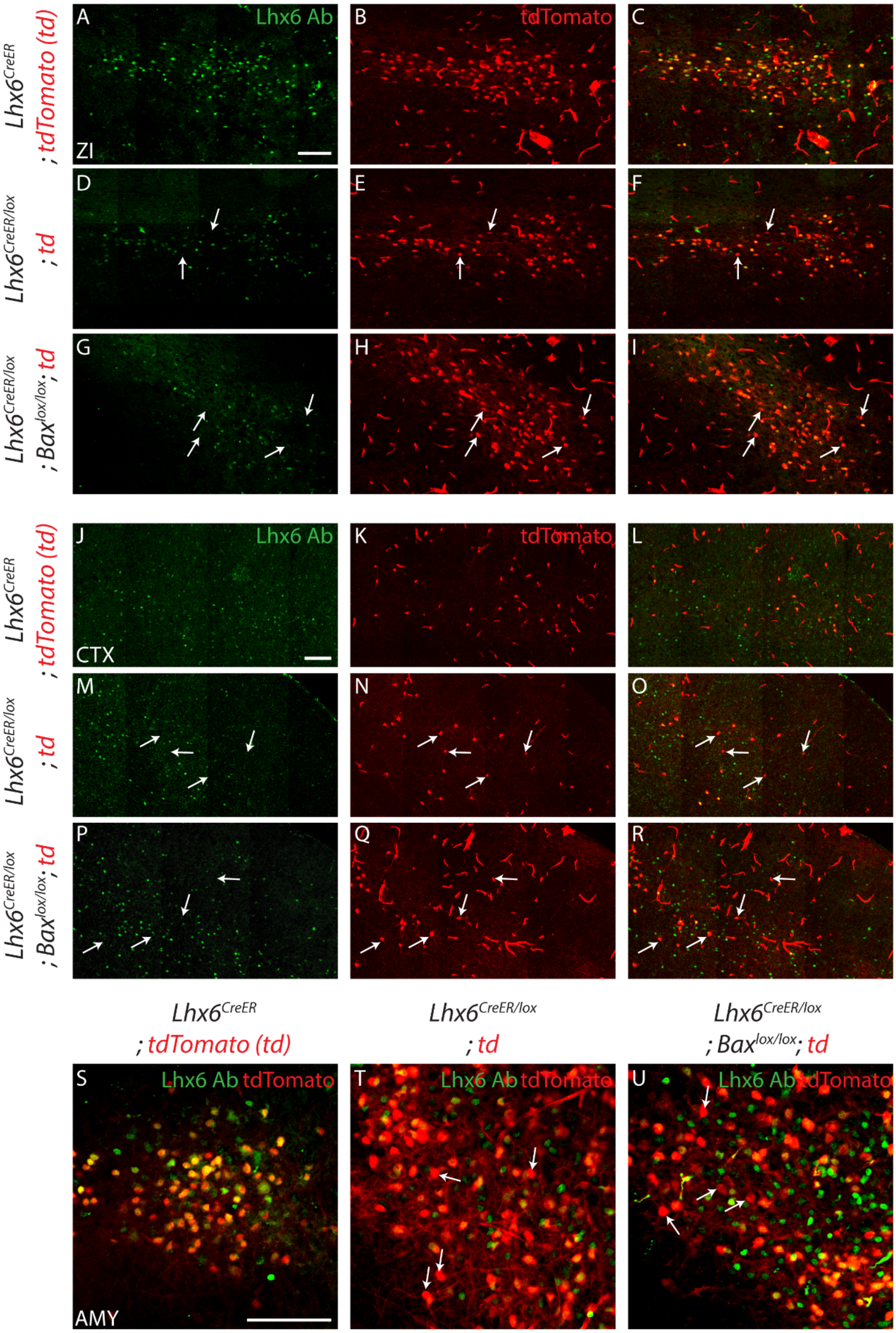

FigS1. Kim, et al.

Supplementary Figure 1. Representative images of 3 genotypes (1. *Lhx6*$^{CreER/+}$*;Ai9* (A-C, J-L, S), 2. *Lhx6*$^{CreER/+}$*; Lhx6*$^{lox/+}$*;Ai9* (D-F, M-O, T), 3. *Lhx6*$^{CreER/+}$*;Lhx6*$^{lox/+}$*;Bax*$^{lox/lox}$*;Ai9* (G-I, P-R, U) in zona incerta (ZI, A-I), cortex (CTX,

J-R), and amygdala (AMY, S-U) with tdTomato (red) and Lhx6 antibody staining (Lhx6 Ab, green). White indicates tdTomato^{+} neurons without Lhx6 expression. Scale bar = 100 μm. 4-OHT was administered between P1 and P5, and animals were collected between P40 and P45.

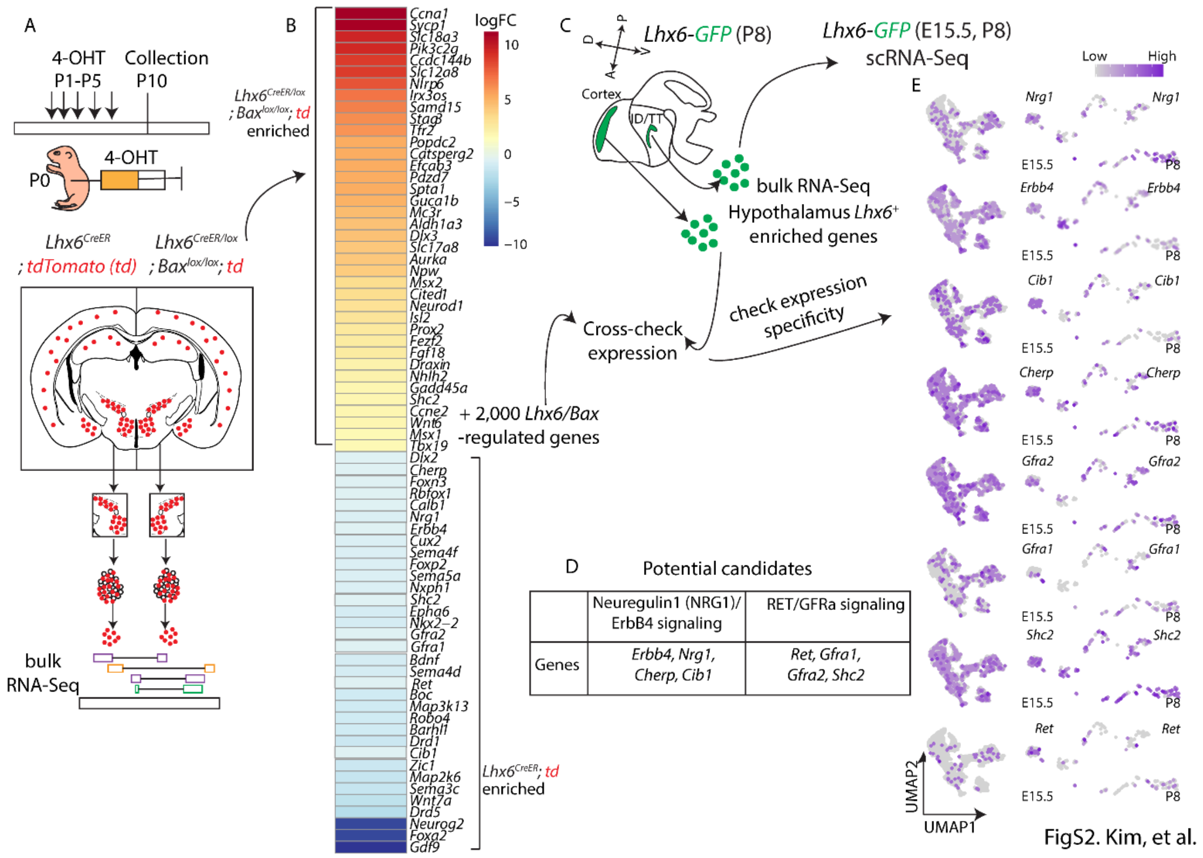

Supplementary Figure 2. Potential candidates that can regulate survival in hypothalamic Lhx6-expressing neurons. (A) Schematic showing bulk RNA-Seq pipeline from $Lhx6^{CreER/+}$;*Ai9* and $Lhx6^{CreER/+}$;$Lhx6^{lox/+}$;$Bax^{lox/lox}$;*Ai9*. (B) A heatmap showing examples of genes (full list in Table. S1) that are enriched in $Lhx6^{CreER/+}$;*Ai9* or $Lhx6^{CreER/+}$;$Lhx6^{lox/+}$;$Bax^{lox/lox}$;*Ai9*. Note upregulation of genes that are involved in cell proliferation (*Ccna1, Aurka*) and neural precursor neurons (*Irx3os, Cited1, Neurod1*). (C) Schematic showing bulk RNA-Seq from P8 *Lhx6-GFP* cortex and hypothalamus, and scRNA-Seq from E15.5 and P8 *Lhx6-GFP* hypothalamus. (D) Potential candidate genes controlling cell survival can be regulated by *Lhx6* in hypothalamic Lhx6-expressing neurons: Neuregulin-ErbB4 signaling and Gdnf signaling pathways. (E) UMAP plots showing that genes in D are robustly expressed in E15.5 and P8 hypothalamus Lhx6-expressing neurons from the *Lhx6-GFP* scRNA-Seq dataset.

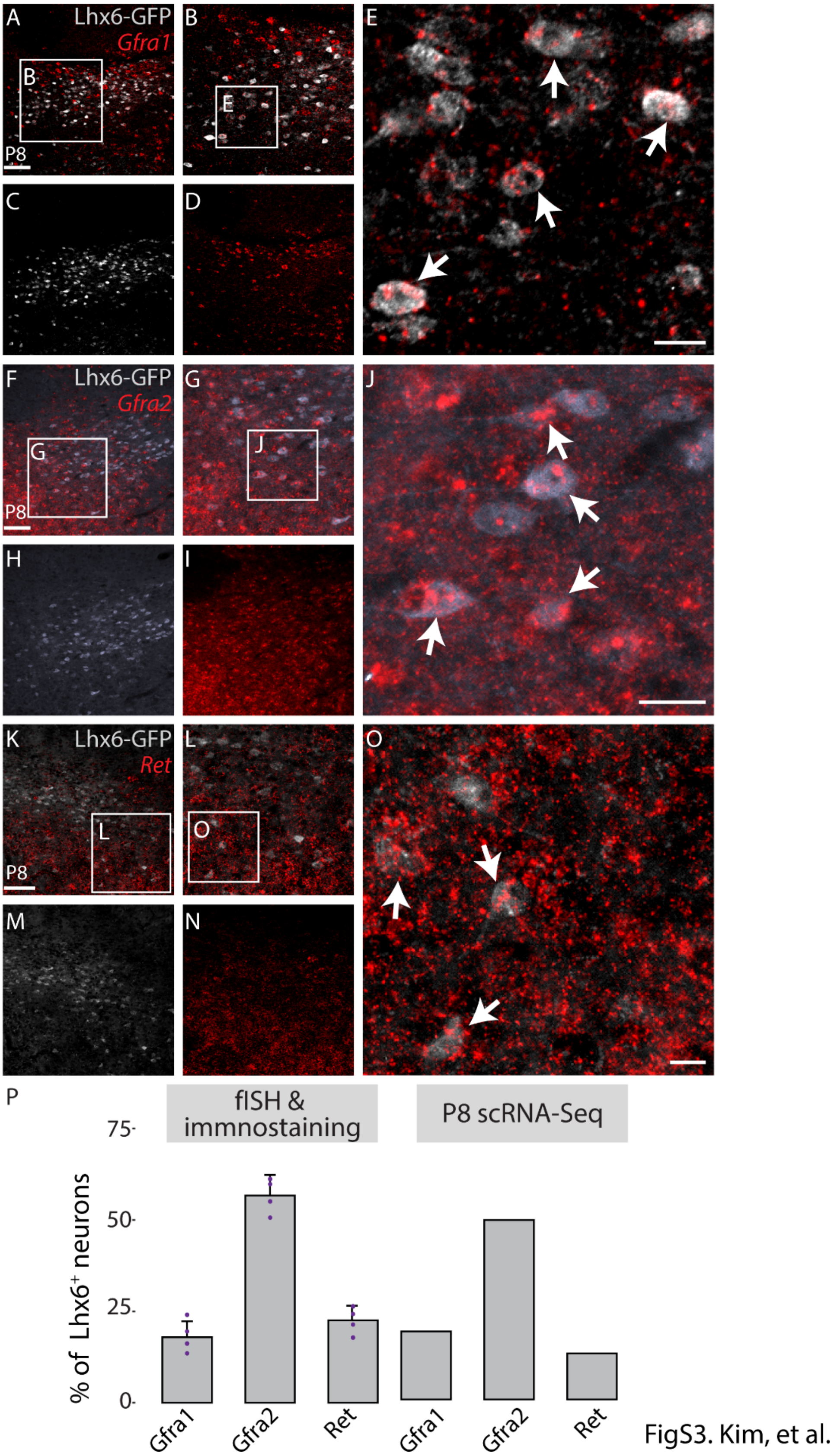

Supplementary Figure 3. Representative images of *Gfra1* (red, A-E), *Gfra2* (red, F-J), *Ret* (red, K-O) in Lhx6-expressing neurons at P8 in the *Lhx6-GFP* line (grey). White arrows in E, J, O show GFP⁺ and *Gfra1*⁺ (E,), *Gfra2*⁺ (J), *Ret*⁺ (O). Scale bar =

100 µm (A, F, K), 15 µm (E, J, O). (P) A bar graph showing the percentage of *Gfra1⁺/Gfra2⁺/Ret⁺* Lhx6-expressing neurons from 1) fISH (*Gfra1/Gfra2/Ret*, Red) and immunostaining of GFP in *Lhx6-GFP* line (grey) (left), and 2) P8 scRNA-Seq data from flow-sorted hypothalamic GFP^+ neurons from *Lhx6-GFP* mice (right).

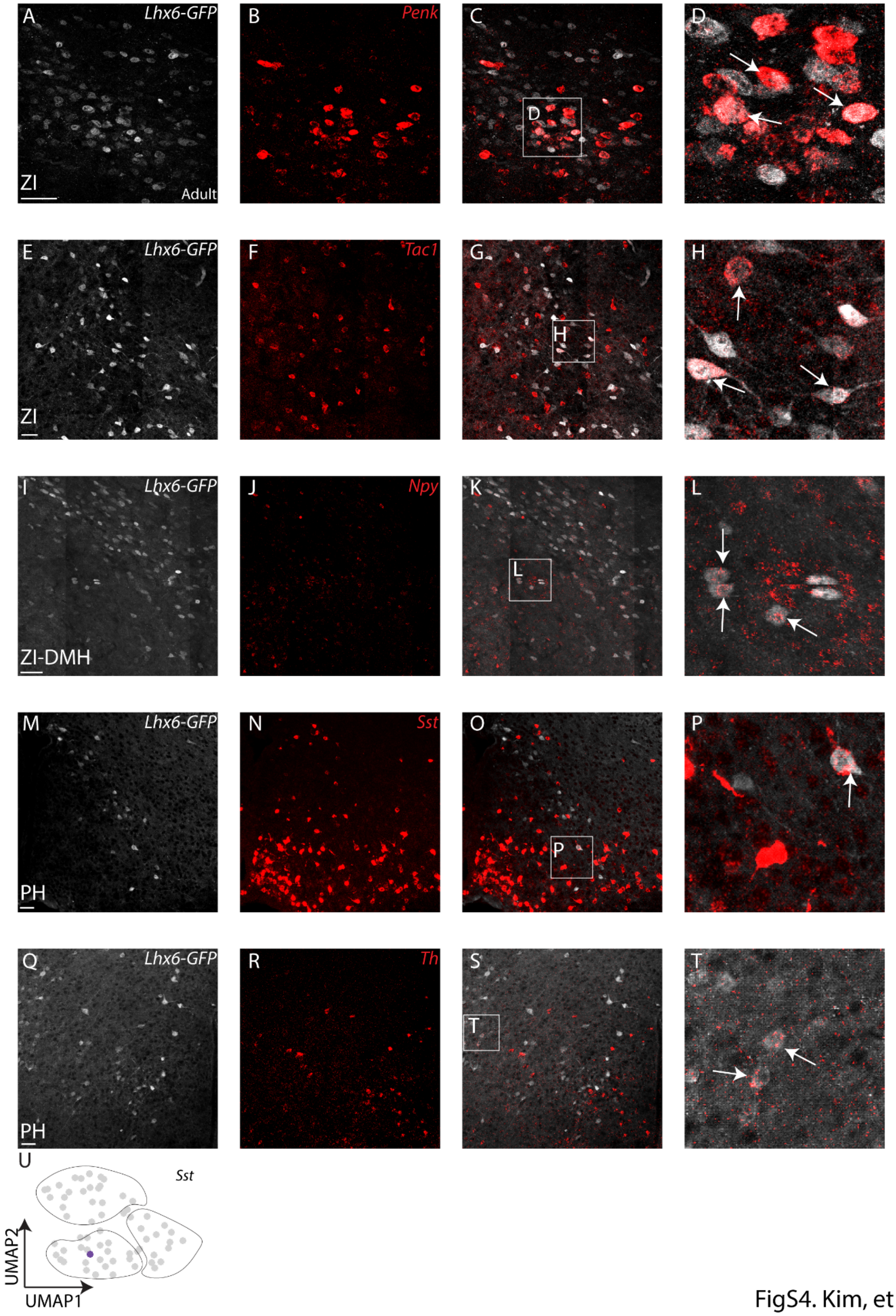

FigS4. Kim, et al.

Supplementary Figure 4. Fluorescent *in situ* hybridization showing *Lhx6-GFP* (grey) with *Penk* (red, A-D), *Tac1* (red, E-H), *Npy* (red, I-L), *Sst* (red, M-P), and *Th*

(red, Q-T). (U) UMAP plot showing *Sst* expression in ID or TT derived Lhx6-expressing neurons at P8. Scale bar = 50 μm.

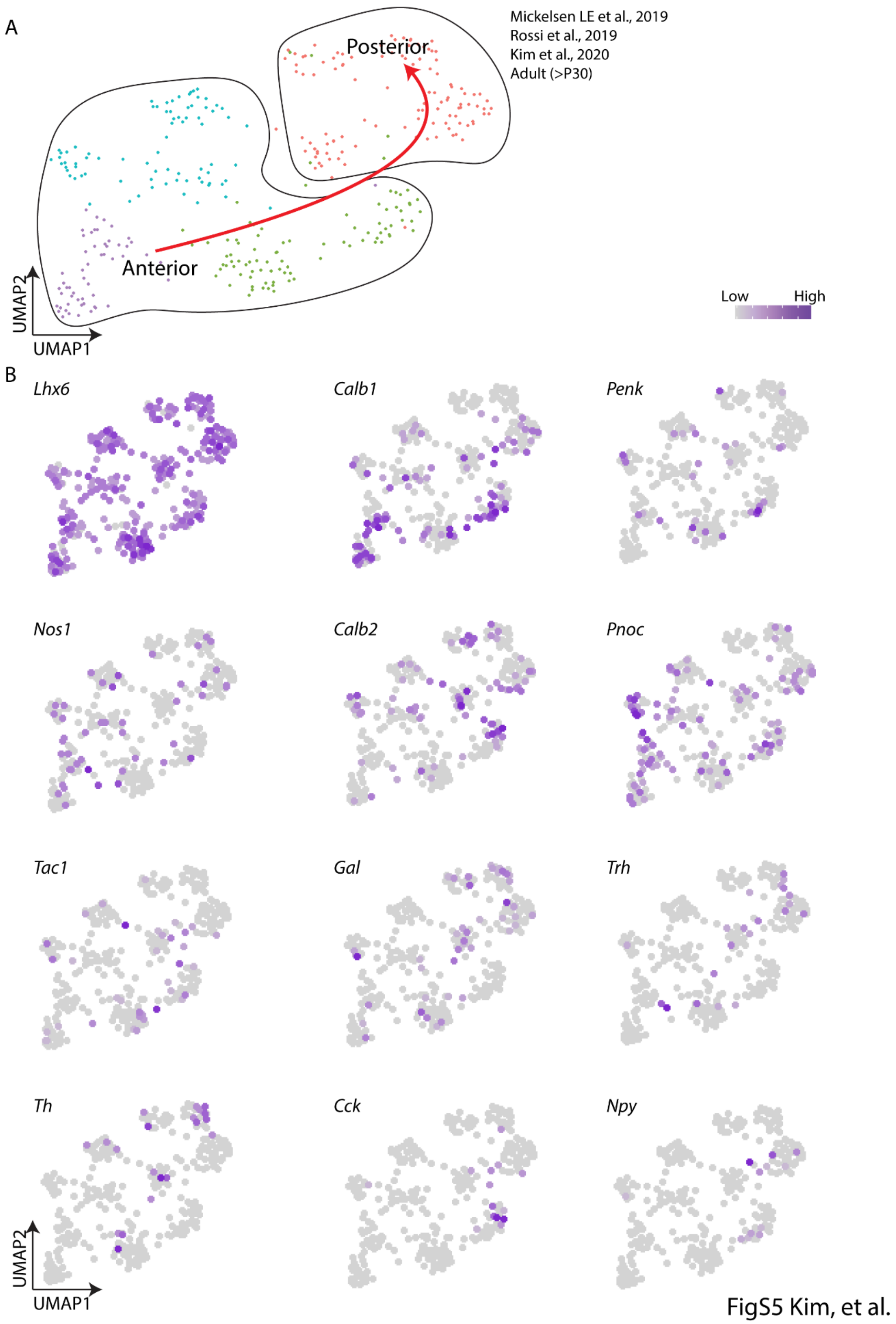

Supplementary Figure 5. (A) UMAP plot showing Lhx6 neurons in adult (P30>) hypothalamus with the anterior-posterior distribution. (B) UMAP plot showing the

expression of neuropeptide and neurotransmitters in adult Lhx6 neurons. Data from [1–3].

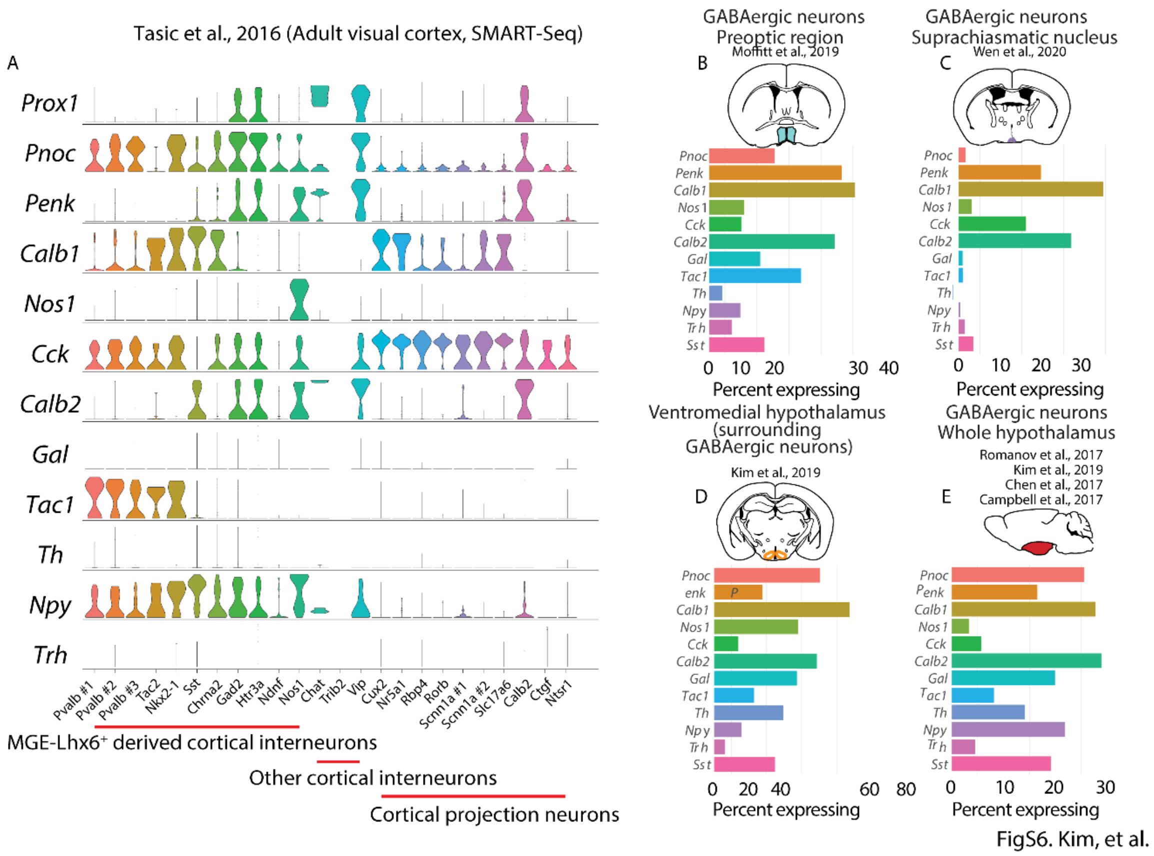

Supplementary Figure 6. (A) Violin plots showing gene expression in visual cortical neurons of key neuropeptides and transmitters that are expressed in hypothalamic Lhx6-expressing neurons. Data from [4]. (B-E) Neuropeptides and neurotransmitters that are enriched in hypothalamic Lhx6-expressing neurons are widely expressed across GABAergic neurons of the hypothalamus - data from the preoptic area (B)[5], suprachiasmatic nucleus (C)[5,6], ventromedial hypothalamus (D)[7], and whole hypothalamus (E)[3,8–10].

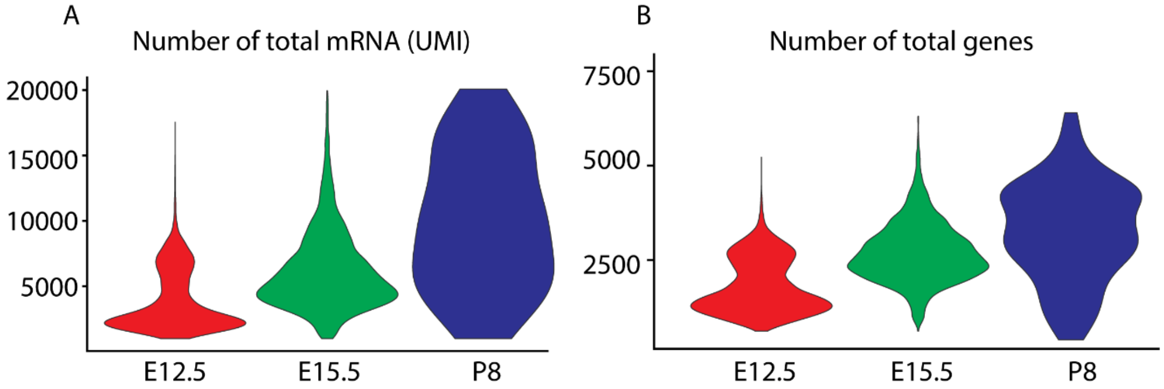

FigS7. Kim, et al.

Supplementary Figure 7. Violin plots showing the number of total mRNAs (A) and the number of total genes (B) in E12.5, E15.5, and P8 *Lhx6-GFP* scRNA-Seq.

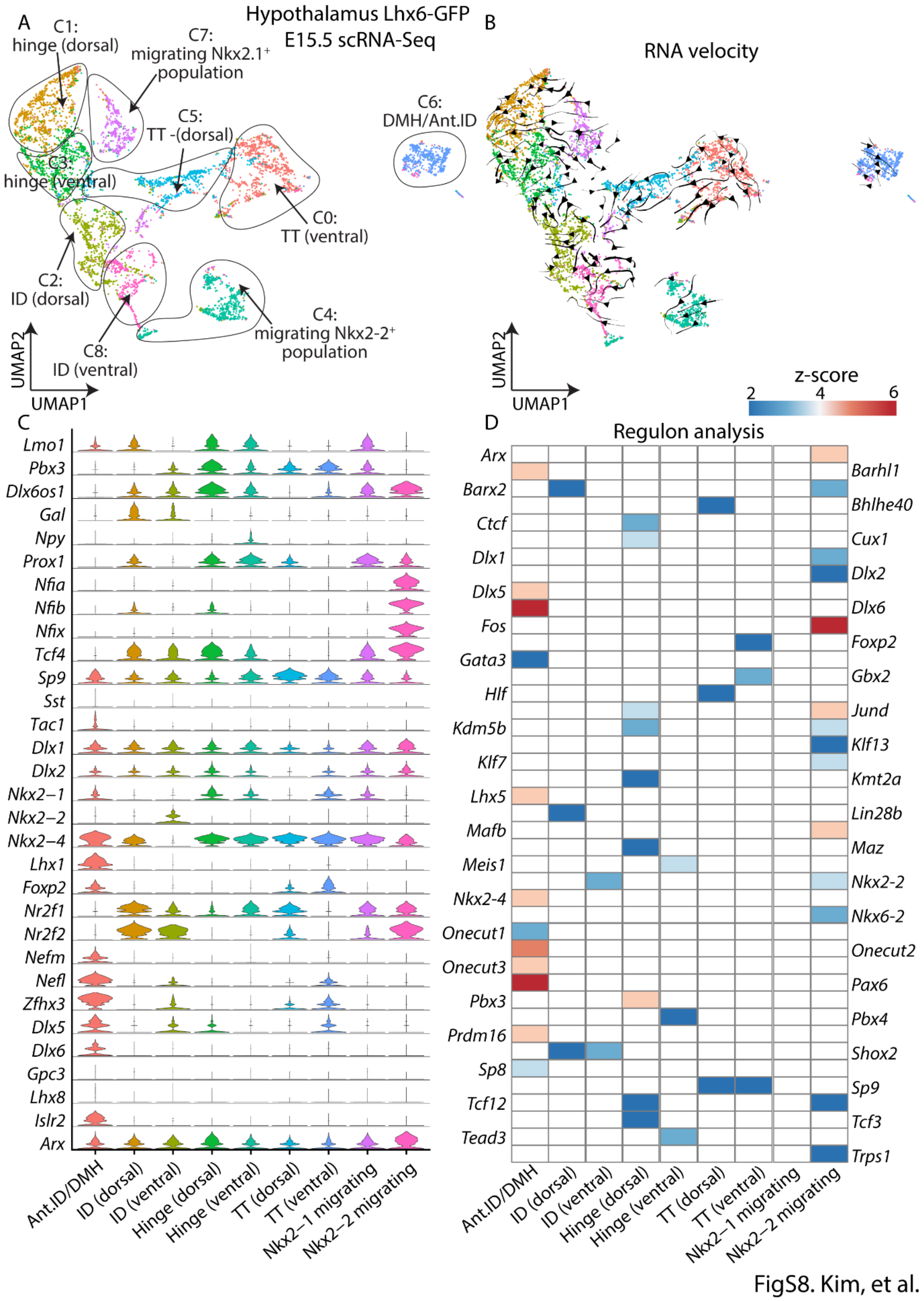

Supplementary Figure 8. (A) UMAP plot showing different Lhx6-expressing hypothalamic regions at E15.5. (B) UMAP plot with RNA velocity trajectories. (C) Violin plots showing expression of key transcription factors (and other genes) that

are highly expressed in individual domains. (D) A heatmap showing z-scores of significantly differentially expressed regulons between Lhx6-expressing hypothalamic regions. Ant.ID = anterior ID, DMH = dorsomedial hypothalamus.

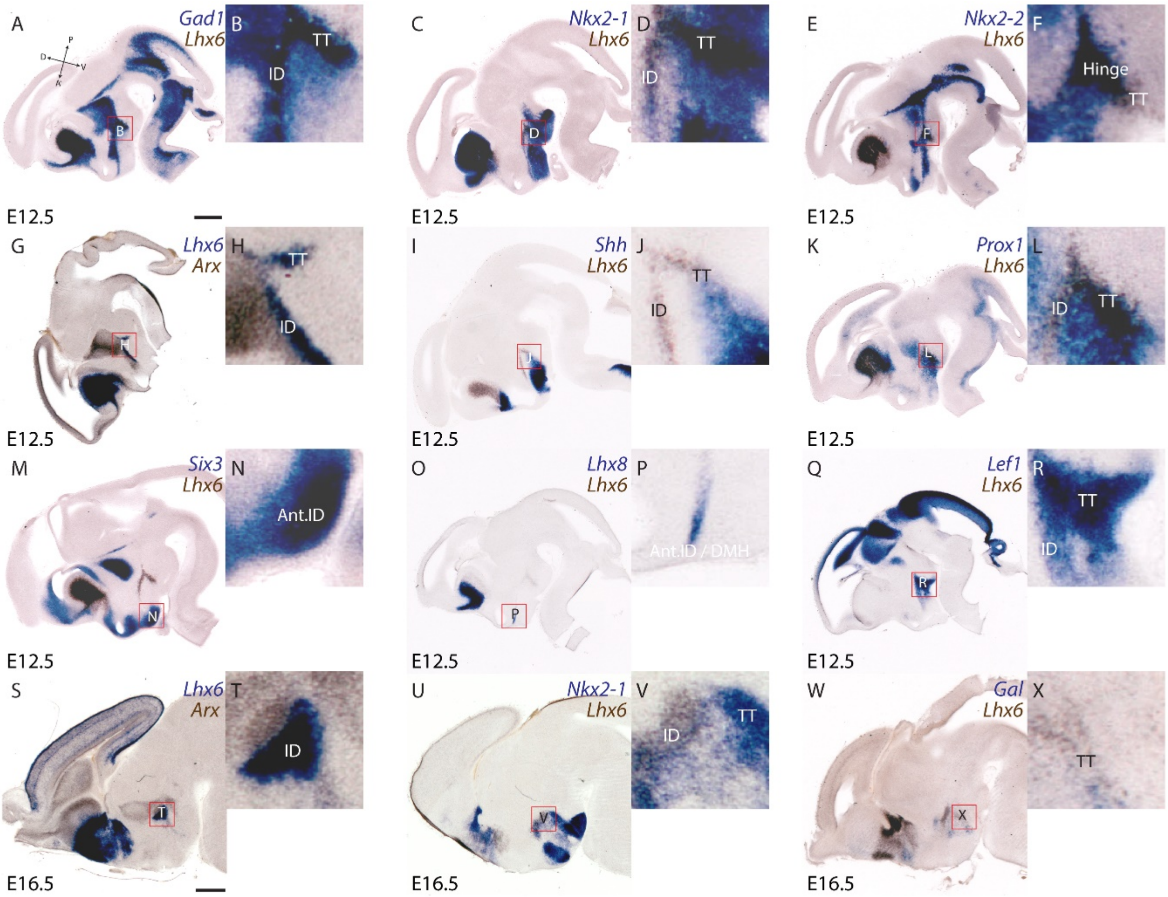

Supplementary Figure 9. *In situ* hybridization showing *Lhx6* with *Gad1* (A, B), *Nkx2-1* (C, D, U, V), *Nkx2-2* (E, F), *Arx* (G, H, S, T), *Shh* (I, J), *Prox1* (K, L), *Six3* (M, N), *Lhx8* (O, P), *Lef1* (Q, R), *Gal* (W, X) at E12.5 (A-R) and E16.5 (S-X), shown in sagittal planes. Scale bar = 0.45 mm (A-R), 0.6 mm (S-X) .

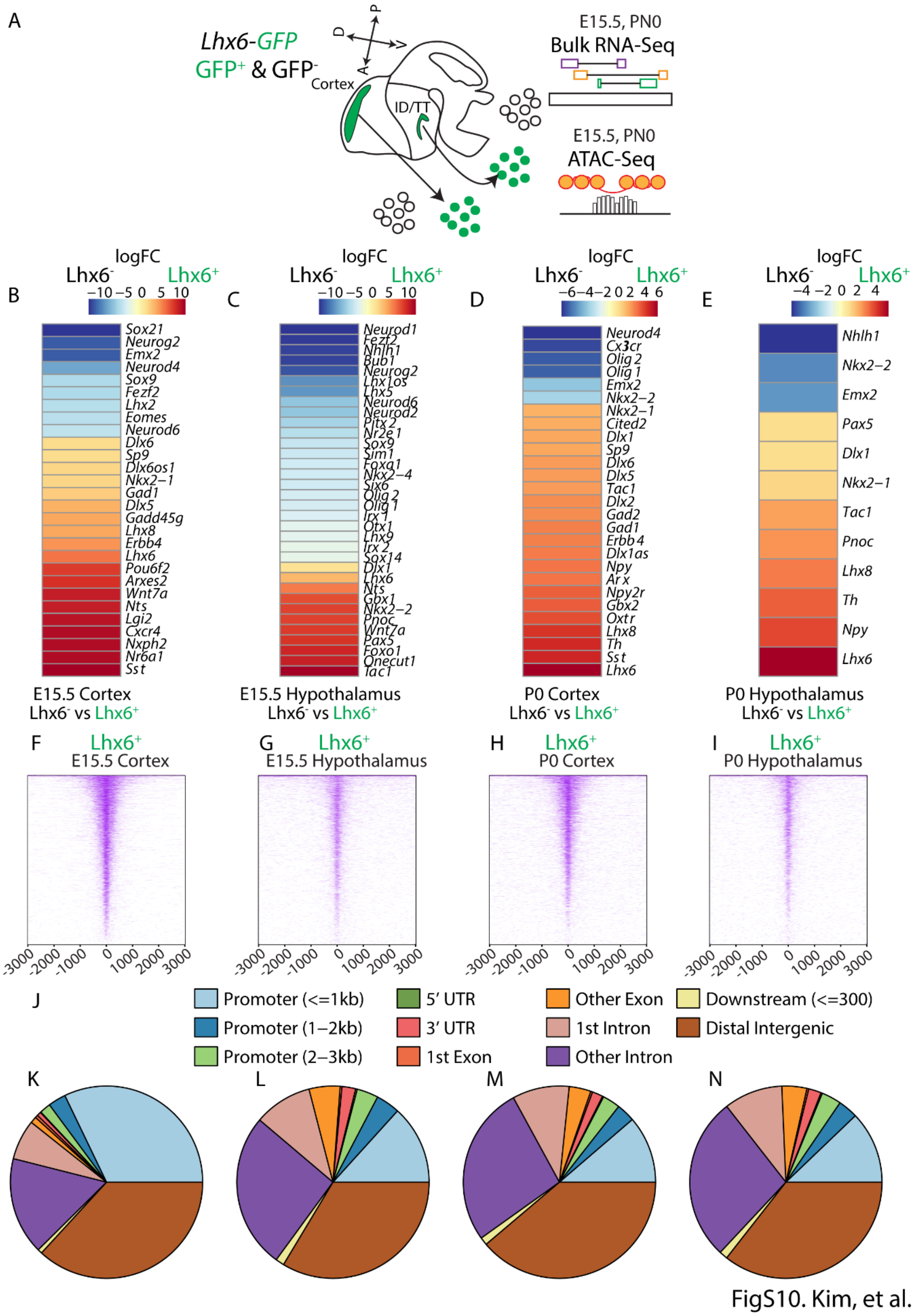

Supplementary Figure 10. (A) Schematic showing bulk ATAC-Seq pipeline from flow-sorted *Lhx6-GFP*+ neurons of the cortex and hypothalamus at E15.5 and P0. (B-E) Heatmap showing examples of genes (full list in Table. S2-S3) that are enriched

in *Lhx6-GFP*$^+$ neurons compared to *Lhx6-GFP*$^-$ neurons of the cortex and hypothalamus at E15.5 and P0. (F-I) Heatmap showing open chromatin regions in E15.5 (F, G), P0 (H, I), cortex (F, H), hypothalamus (G, I). (J) Legends for pie graphs in (K-N). (K-N) Distribution of open chromatin regions in E15.5 (K, L), P0 (M, N), cortex (F, H), hypothalamus (G, I).

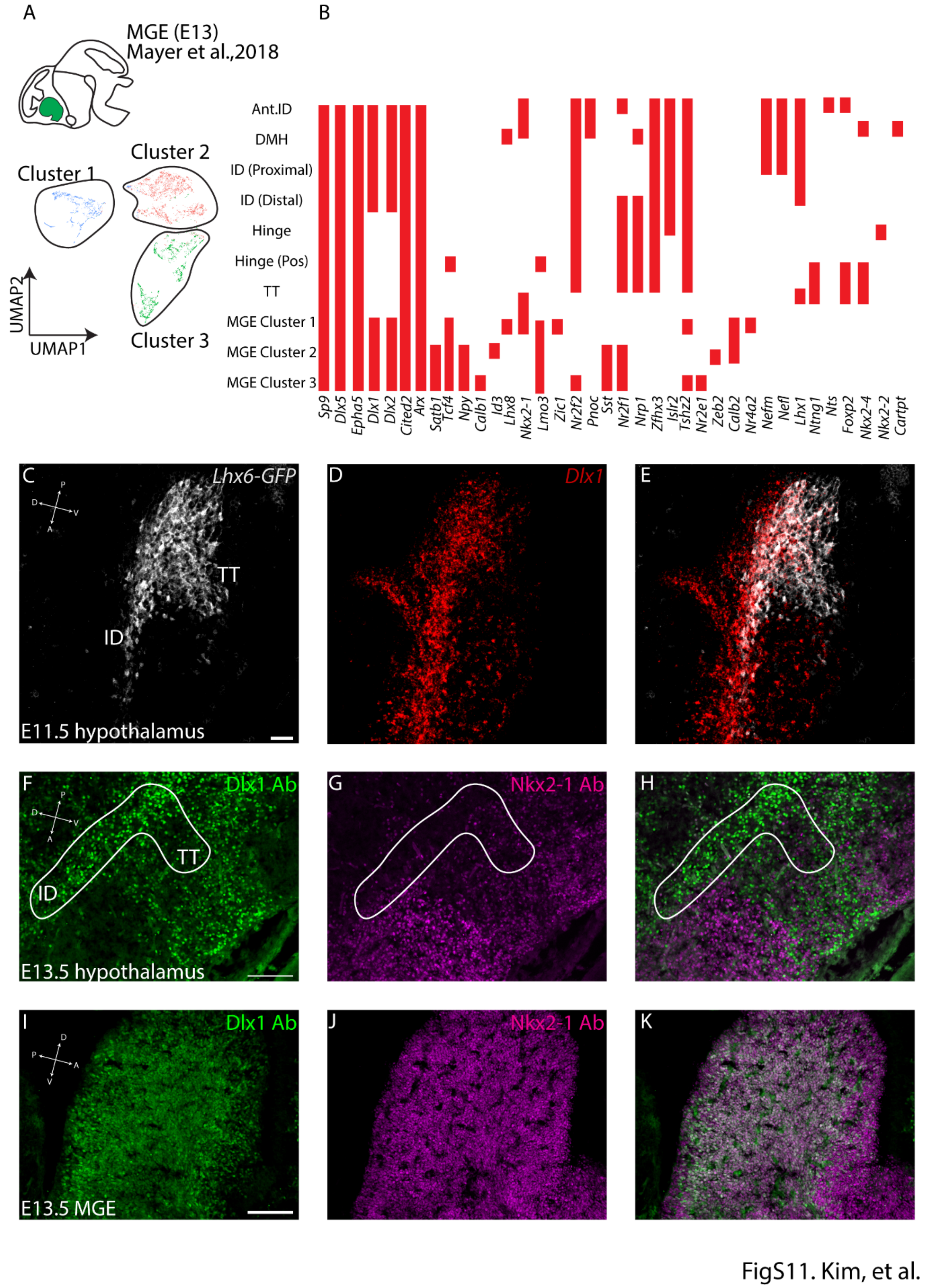

Supplementary Figure 11. (A) UMAP plot showing different Lhx6$^+$ medial ganglionic eminence (MGE) regions at E13.5. Data from [11]**.** (B) Graphs showing presence (red bars) of key genes that are expressed in hypothalamic and/or MGE LHx6$^+$

populations. Note a lack of overlap in gene expression profiles between hypothalamus MGE Lhx6$^{+.}$ populations. (C-E) fISH showing GFP expression in *Lhx6-GFP* line at E11.5 (grey) and *Dlx1* (red). Note *Dlx1* covers most of the ID. (F-H) Immunostaining of Dlx1 (green) and Nkx2-1 (magenta) in E13 ID and TT. Note the lack of co-expression Dlx1 and Nkx2-1, and Dlx1/2 and Nkx2-1 expression delineate separate zones in the ID and TT. (I-K) Immunostaining of Dlx1 (green) and Nkx2-1 (magenta) in E13 MGE. Note a high level of co-expression Dlx1 and Nkx2-1. Scale bar = 50 μm.

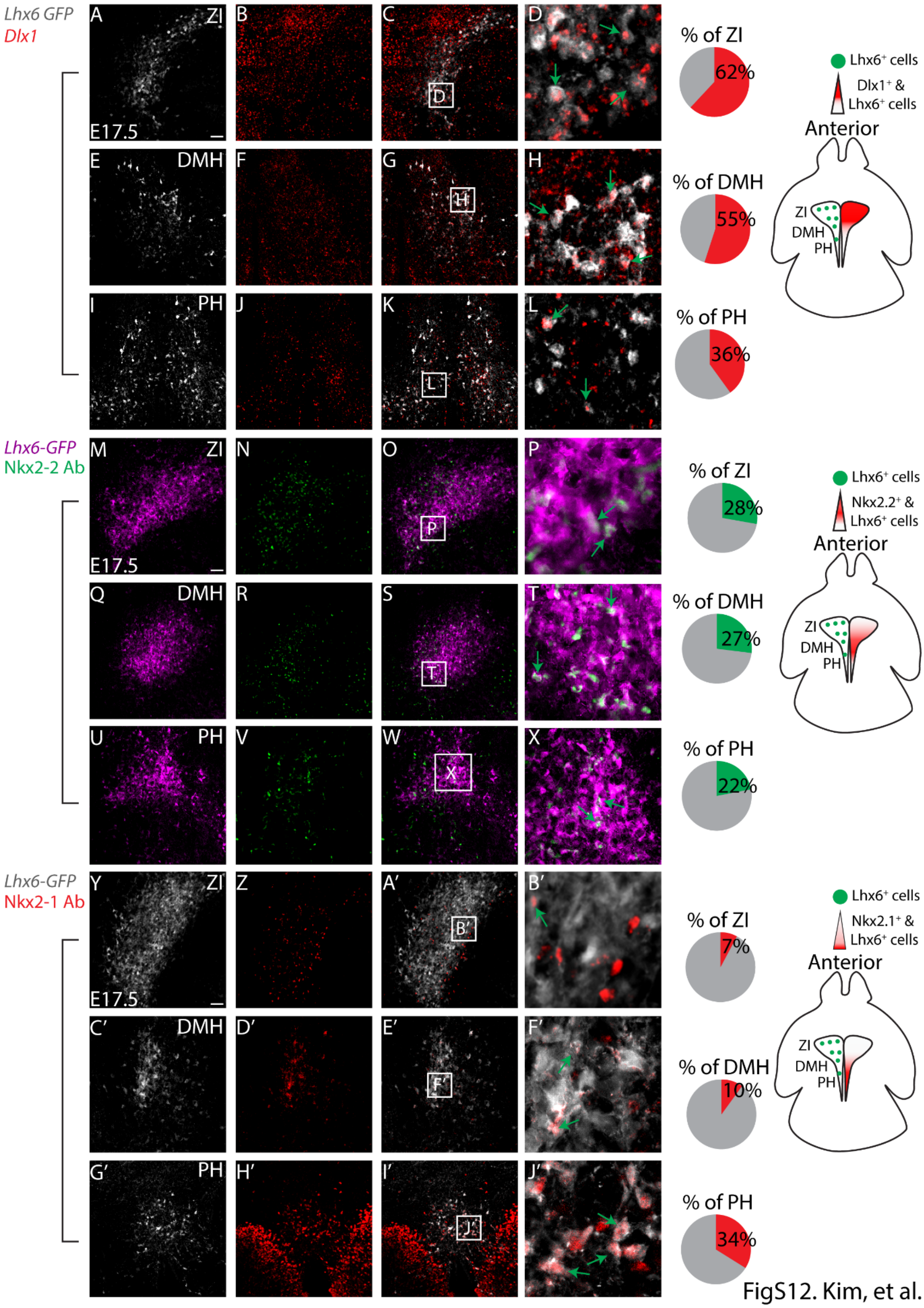

FigS12. Kim, et al.

Supplementary Figure 12. Staining showing distribution and the percentage of *Dlx1* (A-L *Lhx6-GFP* in grey, *Dlx1* in red), *Nkx2-2* (M-X, *Lhx6-GFP* in magenta, Nkx2-2 in green), and *Nkx2-1* (Y-J’, *Lhx6-GFP* in grey, Nkx2-1 in red) in E17.5 zona incerta (ZI,

A-D, M-P, Y-B'), dorsomedial hypothalamus (DMH, E-H, Q-T, C'-F'), and posterior hypothalamus (PH, I-L, U-X, G'-J'). Scale bar = 50 μm.

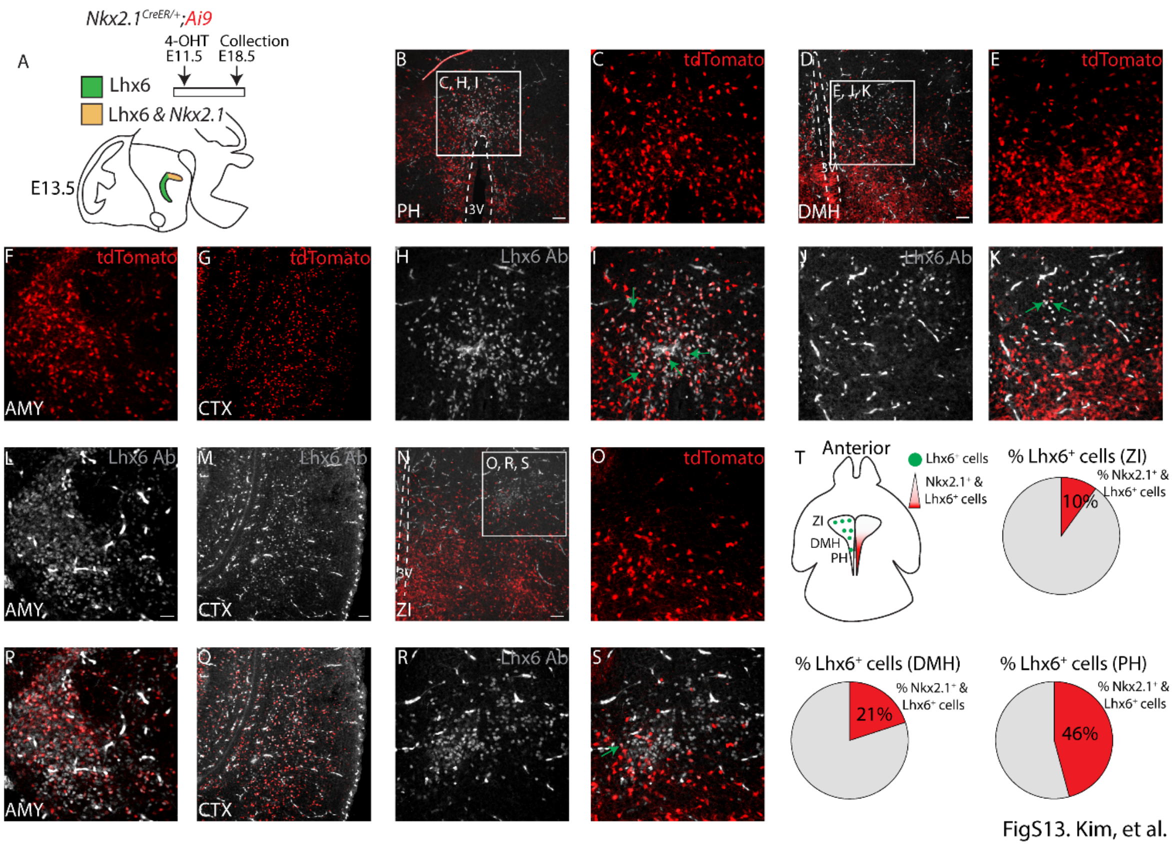

Supplementary Figure 13. Nkx2-1 is required only for the specification of posterior hypothalamic Lhx6-expressing neurons. (A) Schematic showing 4-OHT treatment into E11 *Nkx2-1*$^{CreER/+;}$*Ai9* dams and collection of embryos at E18.5 (top), and schematic showing distribution of *Nkx2-1*$^{-}$*/Lhx6*$^{+}$ in ID (anterior) and *Nkx2-1*$^{+}$*/Lhx6*$^{+}$ in TT (posterior). (B-S) Immunostaining showing Lhx6 (grey) and tdTomato (red, *Nkx2-1*$^{CreER/+;}$*Ai9*) in the amygdala (AMY, F, L, P), cortex (CTX, G, M, Q), posterior hypothalamus (PH, B, C, H, I), dorsomedial hypothalamus (DMH, D, E, J, K), and zona incerta (ZI, N, O, R, ST). Green arrows show co-localization. (T) Schematic showing horizontal mouse brain section highlighting ZI, DMH, PH, and distribution of hypothalamic Lhx6-expressing neurons and Nkx2-1^{+}/Lhx6^{+} neurons are shown (top left). Pie graphs showing the percentage of Nkx2-1^{+}/Lhx6^{+} neurons. Note a posterior bias in the distribution of Nkx2-1^{+}/Lhx6^{+} neurons. Scale bar = 50 µm.

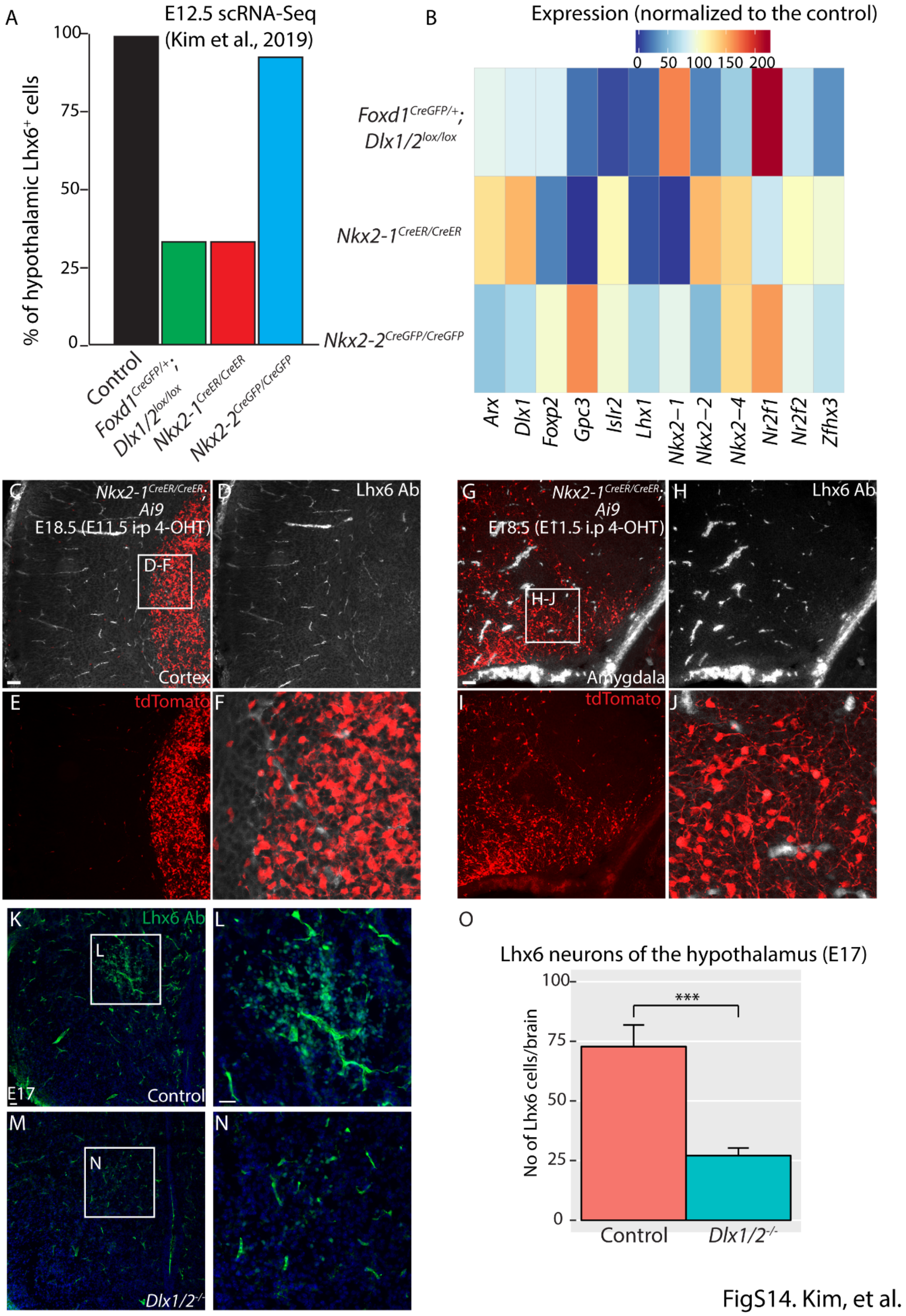

Supplementary Figure 14. (A-B) scRNA-Seq result from [3], *Foxd1*$^{Cre/+}$;*Dlx1/2*$^{lox/lox}$, *Nkx2-1*$^{CreER/CreER}$, *Nkx2-2*$^{CreGFP/CreGFP}$ from hypothalamic *Lhx6*-expressing neurons. (A) Bar graphs showing the percentage of neurons that express *Lhx6* across 4

genotypes. (B) Heatmap showing changes in the percentage of cells expressing individual transcription factors across 4 genotypes. (C-J) *Nkx2-1*$^{CreER/CreER;}$*Ai9* (4-OHT treatment at E11.5, collection at E18.5) showing Lhx6 antibody staining (grey) and tdTomato (red) in the cortex (C-F) and amygdala (G-J). Note the absence of Lhx6-expressing neurons in both brain regions. Scale bar = 50 µm. (K-O) Lhx6 expression (green) in control (K, L) and *Dlx1/2*$^{-/-}$ (M, N) at E17.5 ZI. The number of Lhx6-expressing neurons is shown in (O). ZI = zona incerta. Scale bar = 50 µm. *** $p < 0.05$

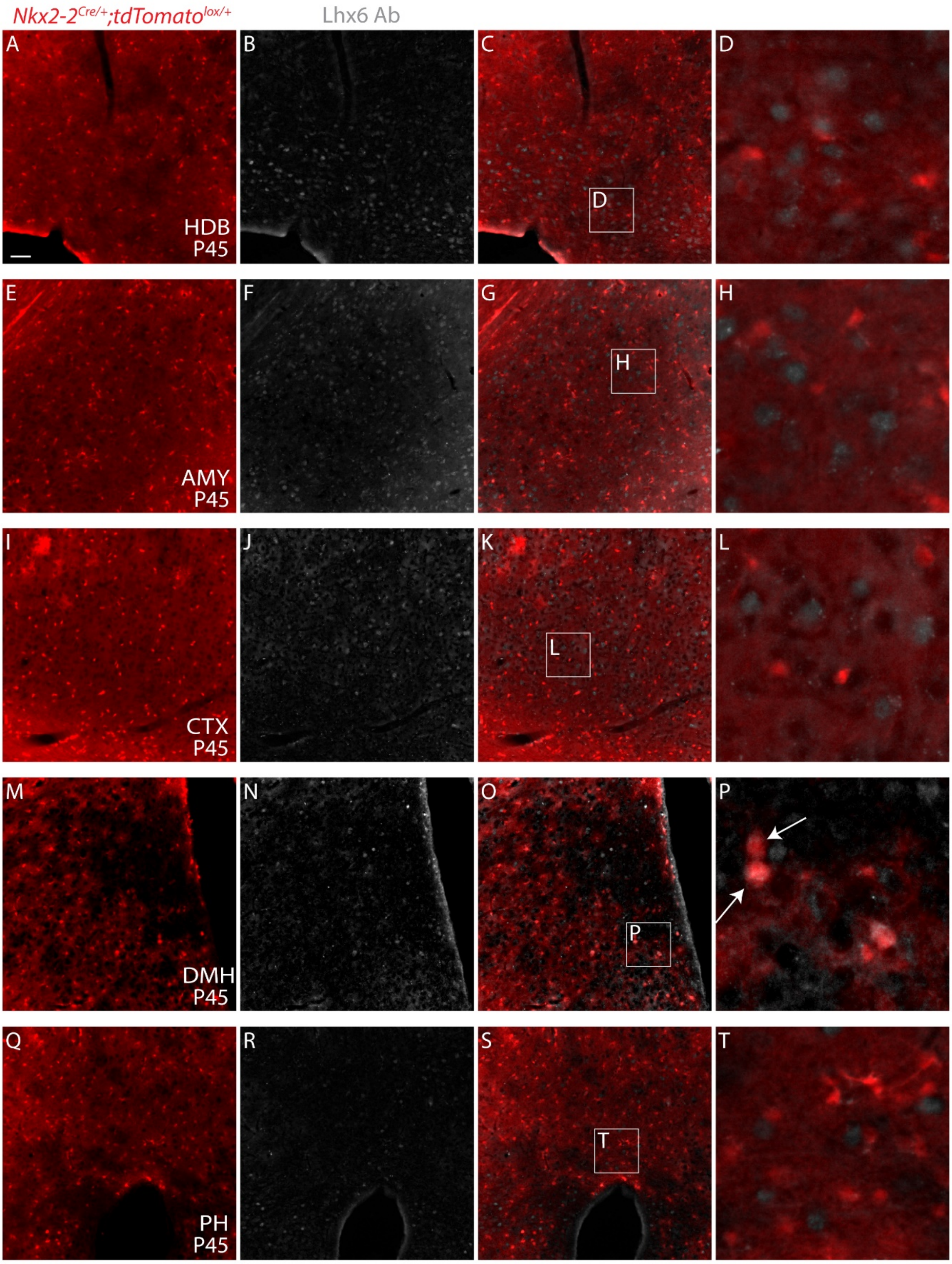

FigS15. Kim, et al.

Supplementary Figure 15. tdTomato expression from *Lhx6*$^{Cre/+;}$*;Ai9* line (red), and Lhx6 antibody staining (grey) in the diagonal band of Broca (HDB, A-D), amygdala

(AMY, E-H), cortex (CTX, I-L), dorsomedial hypothalamus (DMH, M-P), posterior hypothalamus (PH, Q-T).

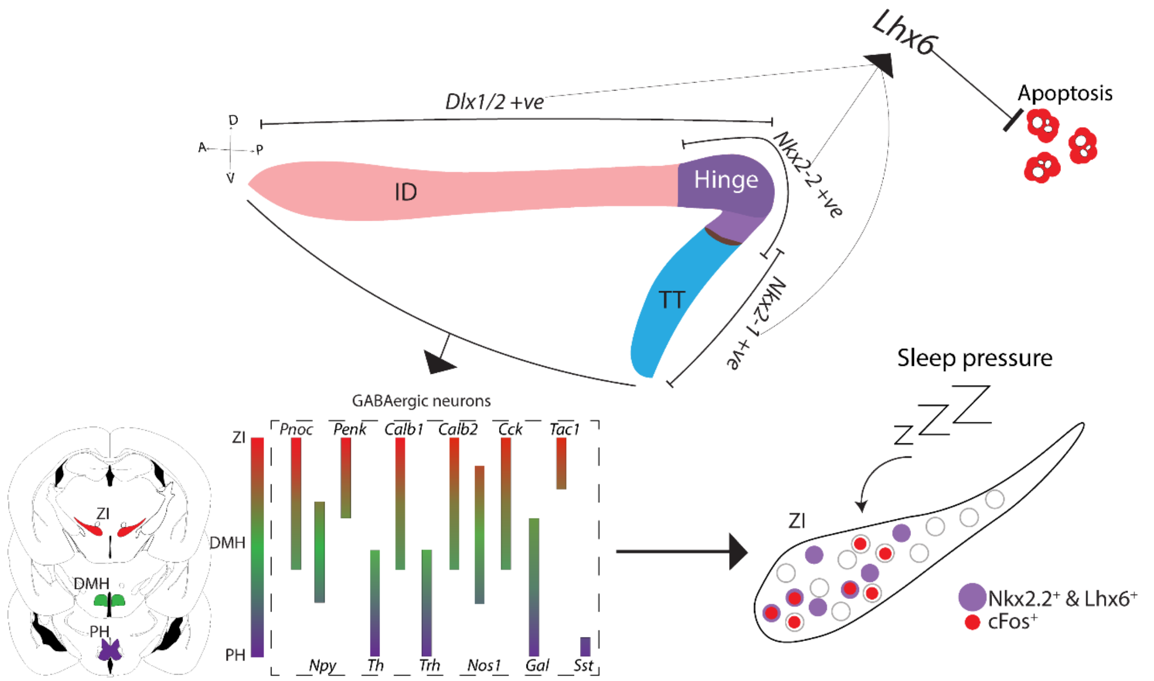

FigS16. Kim, et al.

Supplementary Figure 16. Schematic summary of hypothalamic Lhx6 development and diversity.

Supplementary References:

1. Mickelsen, L. E. *et al.* Single-cell transcriptomic analysis of the lateral hypothalamic area reveals molecularly distinct populations of inhibitory and excitatory neurons. *Nat. Neurosci.* **22**, 642–656 (2019).
2. Rossi, M. A. *et al.* Obesity remodels activity and transcriptional state of a lateral hypothalamic brake on feeding. *Science* **364**, 1271–1274 (2019).
3. Kim, D. W. *et al.* The cellular and molecular landscape of hypothalamic patterning and differentiation. *bioRxiv* (2019) doi:10.1101/657148.
4. Tasic, B. *et al.* Adult mouse cortical cell taxonomy revealed by single cell transcriptomics. *Nat. Neurosci.* **19**, 335–346 (2016).
5. Moffitt, J. R. *et al.* Molecular, spatial, and functional single-cell profiling of the hypothalamic preoptic region. *Science* **362**, (2018).
6. Wen, S. 'ang *et al.* Spatiotemporal single-cell analysis of gene expression in the mouse suprachiasmatic nucleus. *Nat. Neurosci.* **23**, 456–467 (2020).
7. Kim, D.-W. *et al.* Multimodal Analysis of Cell Types in a Hypothalamic Node Controlling Social Behavior. *Cell* vol. 179 713–728.e17 (2019).
8. Romanov, R. A. *et al.* Molecular interrogation of hypothalamic organization reveals distinct dopamine neuronal subtypes. *Nat. Neurosci.* **20**, 176–188 (2017).
9. Chen, R., Wu, X., Jiang, L. & Zhang, Y. Single-Cell RNA-Seq Reveals Hypothalamic Cell Diversity. *Cell Rep.* **18**, 3227–3241 (2017).
10. Campbell, J. N. *et al.* A molecular census of arcuate hypothalamus and median eminence cell types. *Nat. Neurosci.* **20**, 484–496 (2017).
