# Supplementary material for: Gene regulatory networks controlling differentiation, survival, and diversification of hypothalamic Lhx6-expressing GABAergic neurons
Source: Commun Biol. 2021 Jan 21;4:95. doi: 10.1038/s42003-020-01616-7 (PMC7820013; doi:10.1038/s42003-020-01616-7)
Supplement: Supplementary file 2 — Description of Additional Supplementary Files [file 42003_2020_1616_MOESM2_ESM.pdf]

Description of additional supplementary files

File name: Supplementary Data
Description: Excel file containing Supplementary Data 1 – 9.

Supplementary Data 1
Description: Differential gene lists from bulk RNA-Seq between Lhx6CreER/+;Ai9 and Lhx6CreER/+;Lhx6lox/+;Baxlox/lox;Ai9.

Supplementary Data 2
Description: Differential gene expression in P8 hypothalamic Lhx6 scRNA-Seq data.

Supplementary Data 3
Description: Differential gene expression in E12.5 hypothalamic Lhx6 scRNA-Seq data

Supplementary Data 4
Description: Differential gene expression in E15.5 hypothalamic Lhx6 scRNA-Seq data.

Supplementary Data 5
Description: Differential gene lists from bulk RNA-Seq between cortical and hypothalamic Lhx6-GFP+ neurons at E15.5 and P0.

Supplementary Data 6
Description: Differential gene lists from bulk RNA-Seq between Lhx6-GFP+ and Lhx6-GFP- neurons of cortex and hypothalamus at E15.5 and P0.

Supplementary Data 7
Description: Differential peaks from ATAC-Seq between cortical and hypothalamic Lhx6-GFP+ neurons at E15.5.

Supplementary Data 8
Description: Differential peaks from ATAC-Seq between cortical and hypothalamic Lhx6-GFP+ neurons at P0.

Supplementary Data 9
Description: Differential gene expression in E13 MGE Lhx6 scRNA-Seq data.
