# Supplementary material for: Gene regulatory networks controlling differentiation, survival, and diversification of hypothalamic Lhx6-expressing GABAergic neurons
Source: Commun Biol. 2021 Jan 21;4:95. doi: 10.1038/s42003-020-01616-7 (PMC7820013; doi:10.1038/s42003-020-01616-7)
Supplement: Supplementary file 4 — Reporting Summary [file 42003_2020_1616_MOESM4_ESM.pdf]

nature research

Corresponding author(s): Seth Blackshaw

Last updated by author(s): Dec 2, 2020

Reporting Summary

Nature Research wishes to improve the reproducibility of the work that we publish. This form provides structure for consistency and transparency in reporting. For further information on Nature Research policies, see our Editorial Policies and the Editorial Policy Checklist.

Statistics

For all statistical analyses, confirm that the following items are present in the figure legend, table legend, main text, or Methods section.

n/a	Confirmed	
☐	☒	The exact sample size (*n*) for each experimental group/condition, given as a discrete number and unit of measurement
☐	☒	A statement on whether measurements were taken from distinct samples or whether the same sample was measured repeatedly
☐	☒	The statistical test(s) used AND whether they are one- or two-sided<br>*Only common tests should be described solely by name; describe more complex techniques in the Methods section.*
☒	☐	A description of all covariates tested
☐	☒	A description of any assumptions or corrections, such as tests of normality and adjustment for multiple comparisons
☐	☒	A full description of the statistical parameters including central tendency (e.g. means) or other basic estimates (e.g. regression coefficient) AND variation (e.g. standard deviation) or associated estimates of uncertainty (e.g. confidence intervals)
☐	☒	For null hypothesis testing, the test statistic (e.g. *F*, *t*, *r*) with confidence intervals, effect sizes, degrees of freedom and *P* value noted<br>*Give P values as exact values whenever suitable.*
☐	☒	For Bayesian analysis, information on the choice of priors and Markov chain Monte Carlo settings
☐	☒	For hierarchical and complex designs, identification of the appropriate level for tests and full reporting of outcomes
☒	☐	Estimates of effect sizes (e.g. Cohen's *d*, Pearson's *r*), indicating how they were calculated

Our web collection on statistics for biologists contains articles on many of the points above.

Software and code

Policy information about availability of computer code

Data collection	scRNA-Seq (10x v3.0) of flow-sorted GFP+ neurons from the hypothalamus of Lhx6-eGFP line at E12.5, E15.5 and P8, bulk RNA-Seq of flow-sorted GFP+ neurons from the hypothalamus and cortexof Lhx6-eGFP line at E15.5, P0 and P8; bulk ATAC-Seq of flow sorted GFP+ neurons from the hypothalamus and cortex of Lhx6-eGFP line at E15.5, and P0; bulk RNA-Seq of flow sorted tdTomato+ neurons from the hypothalamus at P10
Data analysis	scRNA-Seq datasets were analyzed using the common bioinformatic pipelines. Seurat was used to cluster cells. Scanpy and scVelo were used for RNA velocity analysis, and pySCENIC was used for regulon analysis. Bulk RNA-Seq datasets were analyzed using the common bioinformatic pipelines (Star -> RSEM -> EdgeR). Bulk ATAC-Seq datasets were analyzed using the common bioinformatic pipelines (Bowtie2 -> MAC2 -> Homer).

For manuscripts utilizing custom algorithms or software that are central to the research but not yet described in published literature, software must be made available to editors and reviewers. We strongly encourage code deposition in a community repository (e.g. GitHub). See the Nature Research guidelines for submitting code & software for further information.

Data

Policy information about availability of data

All manuscripts must include a data availability statement. This statement should provide the following information, where applicable:

- Accession codes, unique identifiers, or web links for publicly available datasets
- A list of figures that have associated raw data
- A description of any restrictions on data availability

All sequencing data are available on GEO, GSE150687.

Field-specific reporting

Please select the one below that is the best fit for your research. If you are not sure, read the appropriate sections before making your selection.

[x] Life sciences [ ] Behavioural & social sciences [ ] Ecological, evolutionary & environmental sciences

For a reference copy of the document with all sections, see nature.com/documents/nr-reporting-summary-flat.pdf

Life sciences study design

All studies must disclose on these points even when the disclosure is negative.

Sample size	All staining were performed in triplicates, with tissues from at least 2 different litters. RNA-Seq and ATAC-Seq were ran in duplicates.
Data exclusions	No test samples were excluded from analysis.
Replication	All relevant experimental data is included in this publication. There are no additional unpublished replication results.
Randomization	Animals were assigned randomly to experimental groups for sequencing collection where relevant.
Blinding	All staining (in situ hybridization, RNAScope, immunostaining) were performed blinded.

Reporting for specific materials, systems and methods

We require information from authors about some types of materials, experimental systems and methods used in many studies. Here, indicate whether each material, system or method listed is relevant to your study. If you are not sure if a list item applies to your research, read the appropriate section before selecting a response.

Materials & experimental systems

n/a	Involved in the study	
[ ]	[x]	Antibodies
[x]	[ ]	Eukaryotic cell lines
[x]	[ ]	Palaeontology and archaeology
[ ]	[x]	Animals and other organisms
[x]	[ ]	Human research participants
[x]	[ ]	Clinical data
[x]	[ ]	Dual use research of concern

Methods

n/a	Involved in the study	
[x]	[ ]	ChIP-seq
[x]	[ ]	Flow cytometry
[x]	[ ]	MRI-based neuroimaging

Antibodies

Antibodies used	All antibodies used in this are commercially available and product detail is included in the method section.
Validation	All staining antibodies were previously validated and expression patterns were cross-referenced to scRNA-Seq dataset and the Allen Brain Atlas and Genepaint dataset.

Animals and other organisms

Policy information about studies involving animals; ARRIVE guidelines recommended for reporting animal research

Laboratory animals	Mice of both sexes were used in this study for sequencing.
Wild animals	No wild animals were used in this study.
Field-collected samples	No field-collected samples were used in this study.
Ethics oversight	All experimental animal procedures were approved by the Johns Hopkins University Institutional Animal Care and Use Committee.

Note that full information on the approval of the study protocol must also be provided in the manuscript.
